# Advancing multimodal medical image fusion: an adaptive image decomposition approach based on multilevel Guided filtering

**DOI:** 10.1098/rsos.231762

**Published:** 2024-04-10

**Authors:** Shiva Moghtaderi, Mokarrameh Einlou, Khan A. Wahid, Kiven Erique Lukong

**Affiliations:** ^1^ Department of Electrical and Computer Engineering, University of Saskatchewan, Saskatoon, Saskatchewan S7N 5A9, Canada; ^2^ Department of Biochemistry, Microbiology and Immunology, University of Saskatchewan, Saskatoon, Saskatchewan S7N 5E5, Canada

**Keywords:** medical imaging, multimodal image fusion, salient feature extraction

## Abstract

With the rapid development of medical imaging methods, multimodal medical image fusion techniques have caught the interest of researchers. The aim is to preserve information from diverse sensors using various models to generate a single informative image. The main challenge is to derive a trade-off between the spatial and spectral qualities of the resulting fused image and the computing efficiency. This article proposes a fast and reliable method for medical image fusion depending on multilevel Guided edge-preserving filtering (MLGEPF) decomposition rule. First, each multimodal medical image was divided into three sublayer categories using an MLGEPF decomposition scheme: small-scale component, large-scale component and background component. Secondly, two fusion strategies—pulse-coupled neural network based on the structure tensor and maximum based—are applied to combine the three types of layers, based on the layers' various properties. The three different types of fused sublayers are combined to create the fused image at the end. A total of 40 pairs of brain images from four separate categories of medical conditions were tested in experiments. The pair of images includes various case studies including magnetic resonance imaging (MRI) , TITc, single-photon emission computed tomography (SPECT) and positron emission tomography (PET). We included qualitative analysis to demonstrate that the visual contrast between the structure and the surrounding tissue is increased in our proposed method. To further enhance the visual comparison, we asked a group of observers to compare our method’s outputs with other methods and score them. Overall, our proposed fusion scheme increased the visual contrast and received positive subjective review. Moreover, objective assessment indicators for each category of medical conditions are also included. Our method achieves a high evaluation outcome on feature mutual information (FMI), the sum of correlation of differences (SCD), Qabf and Qy indexes. This implies that our fusion algorithm has better performance in information preservation and efficient structural and visual transferring.

## 1. Introduction

Modern imaging devices offer diverse medical images for clinical screening owing to the ongoing advancements in diverse imaging systems. Various types of medical images are visually examined by a qualified clinician [1]. However, each imaging modality reflects different information about human organs and sick tissue. For example, three imaging methods including magnetic resonance imaging (MRI) , single-photon emission computed tomography (SPECT) and positron emission tomography (PET) are often employed to visualize the abnormalities in the brain. These techniques measure distinct facets of the epileptic process such as structure, metabolism and perfusion. Each technique has its own characteristics, advantages and limitations.

While MRI depicts the brain’s physical anatomical contrast, SPECT demonstrates how it functions. SPECT is proven to reliably evaluate blood flow. MRI does not provide any information on the brain’s function. On the other hand, while PET and SPECT are comparable imaging methods, PET is more expensive. Areas of the brain that are healthy, overactive or underactive can all be seen on SPECT and PET scans. Nevertheless, PET and SPECT have a low spatial resolution.

Commonly, a single modal image lacks adequate data for clinical diagnosis so multimodal medical image can aid medical professionals in developing a more suited treatment strategy [2]. In some cases, a radiologist needs to bring together details from different modalities without sacrificing the original image’s attributes [3]. Additionally, collecting different images from the same patient takes extra space, processing power and time [4]. Recently, as medical imaging continued to advance, multimodal medical image fusion improved remarkably.

Integrating multiple medical images from different imaging procedures is known as medical image fusion which is mainly done to create one image with a large amount of information. Multimodality image fusion combines two or more multimodal input images to generate a more comprehensible and detailed image [5]. Two methods may often be used to combine medical images from various modalities. Hardware device upgrades are one strategy. However, this method is both difficult and costly. Image processing is yet another low-cost and straightforward method [6].

Image fusion is a useful approach to improve computer vision processing since it seeks to combine details from several input images acquired by multimodal imaging equipment [7,8]. Therefore, throughout the process, fusion algorithms should provide strong contrast and integrate the necessary information without generating any artefacts [4]. The aim is to foster medical diagnosis decision-making so the image fusion process consists of a variety of methodologies and research fields, spanning from image processing methods, pattern recognition and computer vision [9–14].

In recent years, many image fusion methods for multimodal input images have been published. The aim is to improve the quality, detail preservation and computational efficiency of the fused image. However, due to their limited ability to process the edges and the textures, the current algorithms often lead to edge artefacts [15]. Properly integrating information from diverse modalities should be managed by including changes in resolution, dynamic range and imaging characteristics. This often includes challenging computer operations and complicated algorithms. On the other hand, optimizing the computational resources is essential to ensuring real-time or close to real-time performance.

Image fusion methods generally involve three main steps: image decomposition, subimage fusion and image reconstruction. The decomposition process, which is the initial stage, divides the source images into several subimages. Some techniques based on the Laplacian redecomposition (LRD) framework are designed to merge multimodal medical images [7]. This method presents a decomposition approach based on Laplacian decision graph to obtain sub-band images. In [16], a multimodal medical image is divided into seven layers using a Gaussian edge-preserving filter and weighted mean curvature filtering. An adaptive decomposition approach is presented in [17] to extract high- and low-frequency components of an image and produce a smoothing and texture sublayer based on Fourier spectrum analysis. The non-subsampled Shearlet transform is another decomposing scheme to acquire low- and high-frequency sub-bands [14]. A two-layer decomposition approach based on joint bilateral filtering is introduced that splits the input image into an energy layer containing substantial pixel intensities and a structure layer [18]. Non-subsampled contourlet transform (NSCT) can also be used as decomposition scheme to decompose the input images into high-pass and low-pass sub-bands. This will generate the detail and structural components of the input image [19].

Inspired by the state-of-the-art decomposition methods, the sublayer containing details and background information are extracted. To accurately produce fused image, fusion rules should be carefully applied to the components from source images. In [20], pulse-coupled neural network (PCNN) are driven by spatial frequency in the NSCT and coefficients in the NSCT domain are chosen as the coefficients for the fused image. A parameter-adaptive PCNN model is used to merge high-frequency bands in [21], in which all the PCNN parameters can be predicted. The majority of machine learning techniques function at the feature and decision levels [22]. In this study [23], authors use deep learning to overcome the fusion problem. They develop a direct mapping across the original images and focus map. This is done by applying CNN which has been trained using the high-quality picture and the blurred variants. An image fusion scheme using convolutional neural network is also presented in [24]. First, weight map is created for the input image by the CNN model. Gaussian pyramid is applied to the weight map to decompose the image. Contrast pyramid is then for the fusion of relatable image components. In the meantime, the local similarity technique in adaptive fusion mode investigates the information of output images. Ultimately, the contrast pyramid restoration yields the fused image.

Fuzzy-based fusion techniques also enhance the precision of target recognition for clinical diagnosis [25]. This work constructs an image fusion technique that combines the benefits of fuzzy entropy and NSCT for multimodal medical images. Finally, according to the decomposition process, inverse decomposition operation is implemented to construct the fused image. An image fusion method based on sparse representation (SR) is reported in [26]. In this article, SR is applied to the texture components, while an energy-based fusion strategy is employed for the cartoon components to uphold the geometric structure information from input images. However, the reduction of computing time remains a significant challenge for SR-based fusion approaches.

To summarize, for improving the diagnostics accuracy, various types of medical images are visually examined by physicians. Each technique has its own characteristics, advantages and limitations. However, collecting different images from the same patient takes extra space, processing power and time. To overcome these issues, multimodal medical image fusion is used. For medical image applications, image fusion is mainly done to compensate for the shortcomings of each of the imaging modalities and to generate one final image with a large amount of information. It is important to have methods that yield strong performance in increasing the visual contrast of the final image. The result images are expected to have less colour distortion and more structural information included. On the other hand, the importance of computational cost efficiency cannot be neglected. Computational efficiency is important in processing large datasets and complex images in a timely manner to ensure that diagnostic procedures are not taking long. As a result, there should be a balance between high-quality, multimodal medical imaging fusion and the computational efficiency of the algorithm.

In the field of medical image fusion, research papers often lean towards either achieving exceptional visual contrast or focusing on optimizing time efficiency in terms of processing speed. It is challenging to find a balance between the two of these variables. In our efforts in this work, we have developed a novel algorithm designed to offer outstanding visual contrast, ensuring that fused medical images are not only visually appealing but also facilitate accurate diagnosis. Simultaneously, we tried to have a relatively time-efficient algorithm in terms of time cost evaluation. This makes our approach applicable for almost real-time medical scenarios.

In this article, we deliver a novel image fusion strategy for brain images. The method used the multilevel Guided edge-preserving filtering (MLGEPF) to decompose the source image into seven sublayers. MLGEPF is implemented by applying Gaussian filter and a modified version of Guided filter with iteratively updated guidance image. We have altered the guidance image from the input image to difference image of the input image and magnitude of gradient, so that the small fluctuations of the input image can be largely smoothed. This will maintain clear edge information and smooth complex small areas in an image. In this decomposition approach, the input image is breaking down into small-scale component (SC), large-scale component (LC) and background component (BC) layers using an edge-preserving Guided filter.

Then, to create three different fused layers, SC, LC and BC layers are combined using a novel gradient domain PCNN based on the structure tensor fusion technique and a maximum-based fusion approach, respectively. In our novel PCNN-based fusion approach, we use the image’s gradient magnitude as the structural tensor. The structure tensor is applied as the input for the PCNN network. We take advantage of this for leveraging the strength and orientation of edges and corners in the images. This approach enables PCNN to highlight important features that lead to a more effective fusion of MRI and PET/SPECT images. The three fused layers are then combined to reconstruct the fused image.

The suggested method and other novel approaches are examined both qualitatively and quantitatively to assess the fusion results. Our proposed technique exhibits better performance compared with current fusion methods in qualitative and quantitative evaluation. In our experiments, a total of 40 sets of medical images from four categories of medical conditions are tested. This article is structured as follows: an in-depth discussion of the suggested decomposition and fusion rule is given in §2. The results and discussions of experiments are demonstrated in §3. The conclusion is covered in §4.

## 2. Proposed method

**Figure 1.**
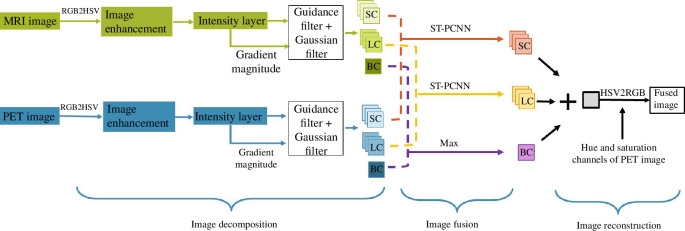
Block design illustrating the proposed medical image fusion technique.

This work proposes an edge-preserving filtering fusion technique that draws inspiration from PCNN fusion and image decomposition schemes. Our multimodal image fusion approach is suggested and described in this section. A visual representation of the suggested model is provided in figure 1. The algorithm consists of three components: image decompression using MLGEPF, image fusion and image reconstruction.

It is worth mentioning that the model starts with hue, saturation, value (HSV) colour space transform on both input images. An inverse HSV transform is then performed to create the final fused image after image pair fusion. The first step would be decomposing the input image pair into seven informative layers through the guidance filter edge-preserving scheme. Next, the BC layers are fused by maximum-based fusion scheme. An Structure tensor (ST)–PCNN fusion approach is performed on the SC and LC layers. After performing the fusion, linear addition is used to create the final fused image.

### 2.1. Image enhancement

Medical imaging devices, including MRI and X-ray machines, generate distortions in the captured images. The variations between organs and tissues in various images may be quite low, resulting in a poor visual effect when viewed through human eye. Because human eyes can only respond to brightness within a particular range [4,27]. Image enhancement is essential in the field of image processing because it optimizes image quality by highlighting useful information and eliminating redundant information [28]. Therefore, by altering the contrast of the source images, the negative effects are minimized.

### 2.2. Image decomposition

**Figure 2.**
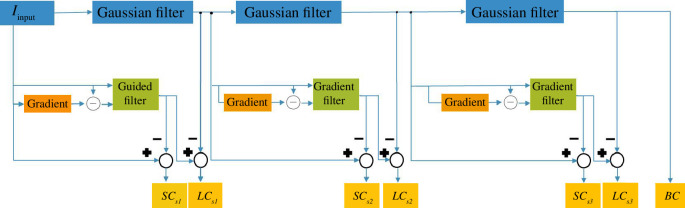
MLGEPF decomposition.

**Figure 3.**
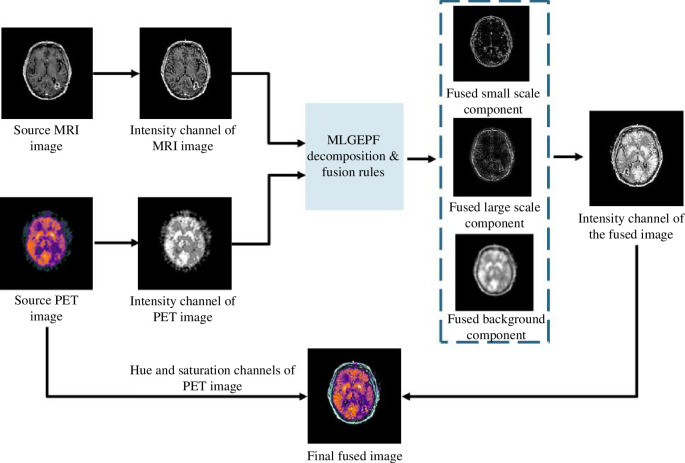
The proposed fusion method’s structure.

The structure diagram of MLGEPF decomposition is displayed in figure 2. It is worth mentioning that the number of decomposition layers must be carefully chosen with the goal of minimizing redundancy. The number of decomposition layers should be chosen after considering the trade-offs between information retention, computational complexity, noise sensitivity and fusion method requirements. Having fewer layers reduces the overall quality. A limited number of layers could risk overlooking subtle details like edges. This is mainly due to the fact that the image decomposition is reliant on the intensity of pixels and the local information. For example, if the image is decomposed into only two components, such as large- and small-scale components, subtle edges might be overlooked during the process. In these situations, the algorithm divides the information based on pixel intensity, and since the model might not capture subtle variations in the image with a limited number of layers, there is a higher chance of missing features. On the other hand, too many layers produces computational and duplication issues [22]. Too many layers may introduce computational challenges and duplication issues, potentially saturating the output with information. Even misleading information may be generated during the decomposition process. Figure 3 depicts the main procedures of the proposed method and the output at each level.

The Gaussian blur feature is obtained by smoothing an image using Gaussian filtering to reduce the amount of details and also the noise level. The Gaussian filter serves as a non-uniform low-pass filter similar to the mean filter, but with a different kernel. The two-dimensional Gaussian filter is used when working with images. It is simply the product of two one-dimensional Gaussian functions.

A conventional approach in image processing is to smooth the image while keeping the structures and edges. Edge-aware filters are popular filters in visual processing. Different methodologies are used to construct edge-aware filters. However, the goal is to maintain only high-contrast edges [29]. Edge-preserving filters include bilateral filter, Guided filter, geodesic filters and weighted median filters. The primary goal of the guidance filter is to efficiently eliminate detailed texture and noise while preserving the edges as much as possible.

**Figure 4.**
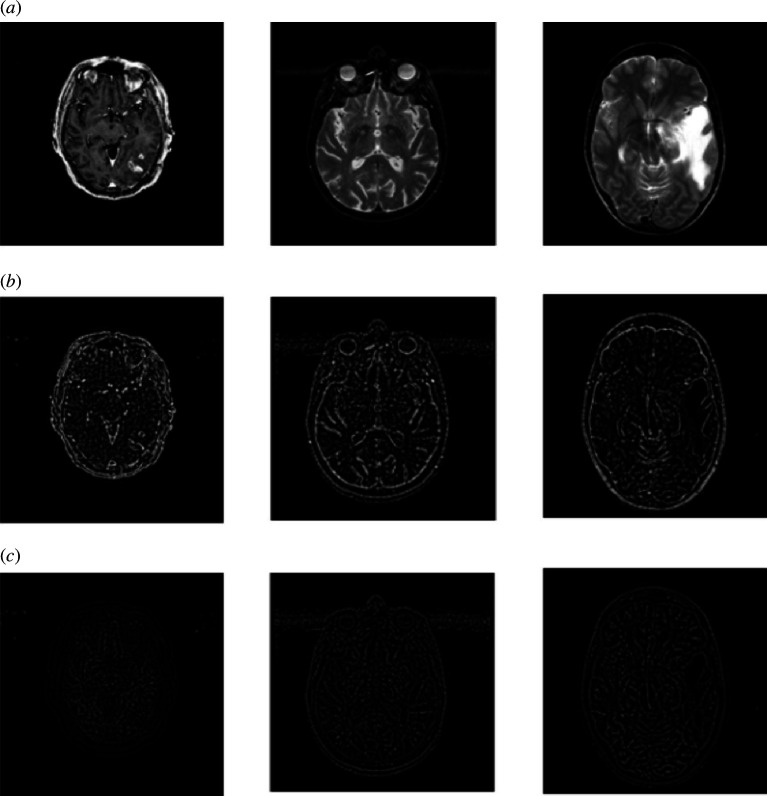
Illustration of the Guided filter output. (*a*) original MRI and PET images. (*b*) SC extracted using the gradient magnitude and the Guided filter. (*c*) SC extracted using the conventional Guided filter.

**Figure 5.**
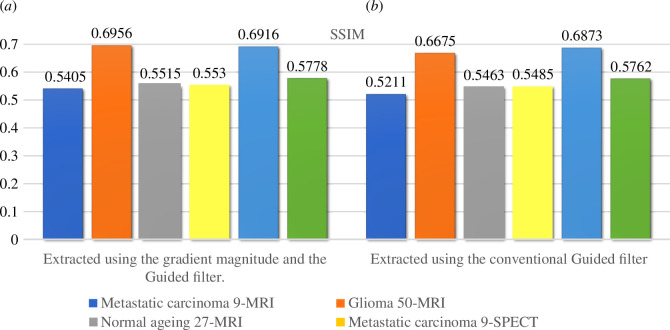
Comparison of SSIM. (*a*) Extracted using the gradient magnitude and the Guided filter. (*b*) Extracted using the conventional Guided filter.

The filtering output determined by the Guided filter takes into account the structures of the guidance image which can be the input image or a different image [30]. Beyond smoothing, the Guided filter has a unique concept: it is capable of transferring the guidance image’s features to the filtering output, useful in applications such as dehazing [30]. The input image is smoothed using the input image itself as the guidance image in the primary framework of Guided image filter to approach the edge preservation effect [30]. Figure 4 illustrates examples of the proposed Guided filter performed on the input images. In this layout, we compared the smoothed images by the original Guided filter (the image itself as the guidance image) and our proposed composition method (difference image of the input image and magnitude of gradient as the guidance image). It can be seen that the significant features retrieved by our method are more than those extracted by the original Guided filter. Structural similarity index (SSIM) comparison of these two categories is reported in figure 5. As can be seen, images extracted using gradient magnitude and the Guided filter have slightly higher SSIM, which means more similarity to the reference image.

**Figure 6.**
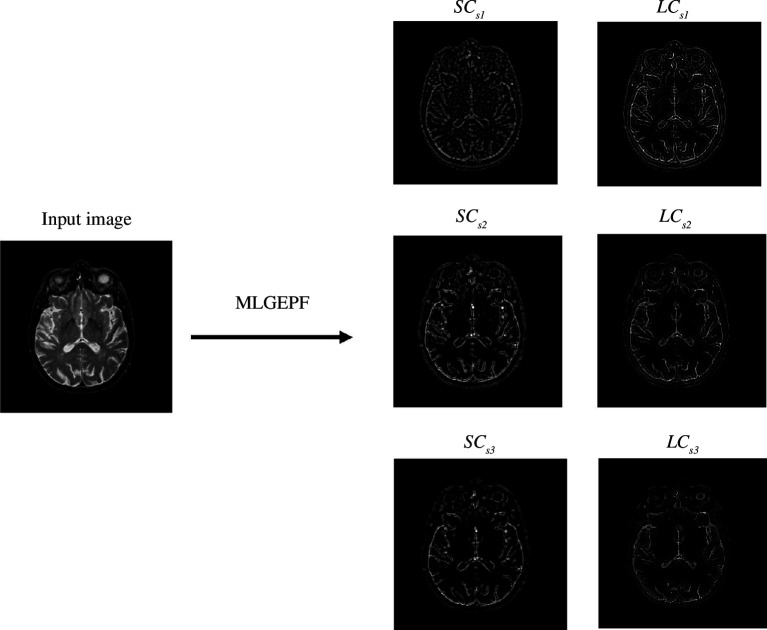
Subimages generated by MLGEPF.

Figure 6 displays the subimages extracted by the proposed MLGEP. Since there are three decomposition layers, the background information is separated from the small-scale components gradually.

In this article, the MLGEPF is done by applying Gaussian filter and Guided filter. The MLGEPF diagram is presented in figure 2. The image components are formed by the filtered outcomes of the *i*th Gaussian filter and the *i*th Guided filter in the block diagram. The three types of layers are represented as SC(i), LC(i) and BC.


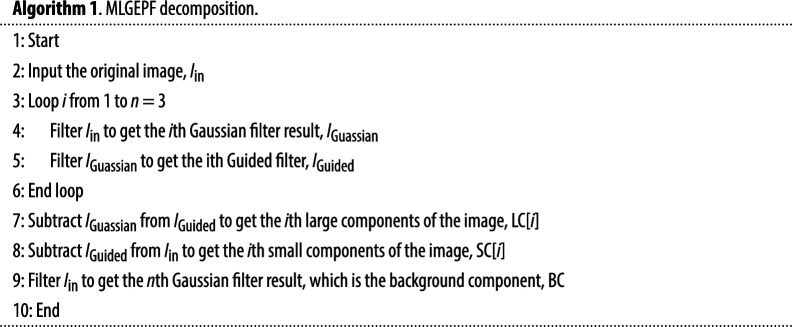


In this work, a new adaptive decomposition strategy is presented through MLGEPF to decompose the input pair images into seven layers. In the next level, the gradient magnitude of the input image is first determined. Image also goes through Guided filter. Then, the filtered image is used as a fresh guidance image in the next iteration for filtering the input image.

So the guidance image is iteratively updated. In other words, we have altered the guidance image from the input image to difference image of the input image and magnitude of the gradient, so that the small fluctuations of the input image can be largely smoothed. Accordingly, the salient features (see figure 4) extracted by our Guided filter are far more than those extracted by the original Guided filter.

### 2.3. Image fusion

Using the decomposition technique described above, the sublayer containing rich details and background information is generated. To accurately produce fused image, fusion rules should be carefully applied to the components from source images. A novel gradient domain PCNN based on the structure tensor and maximum-based fusion strategies are applied on SC, LC and BC.

#### 2.3.1. Pulse-coupled neural network

PCNNs have been widely employed for image processing techniques such as image segmentation and pattern recognition. In the process of image fusion, PCNN is a single-layered two-dimensional array of linked neurons. The network’s number of neurons is equivalent to the total number of pixels in each input image. The pixels in each image have a one-to-one correspondence with neurons. For the stimulus of the neuron, the grey value of the neuron is employed [31]. PCNN has made a significant impact on image fusion since it is not required to do training [16]. It can also effectively quantify the activity level of each pixel. This involves modelling how neurons respond to input stimuli. PCNN processes the pixel activity based on local and global interactions in each iteration. The network’s pulse synchronization mechanism enhances its ability to capture significant features of the input images. This approach contributes to the effectiveness of PCNN for image fusion applications [21]. Also PCNN enables quick and effective computations, which is an important feature, especially for medical image fusion tasks [32].

We have applied PCNN to improve the spatial correlation of the comparable layers. Before the fusion is processed, the structure tensor salient (STS) operator is also applied to layers.

#### 2.3.2. Structure tensor

MRI images contain more strong edges and structural features, while PET/SPECT images contain more colour information. MRI-SPECT and MRI-PET fused images should retain both structure and colour information. In order to prevent ‘unnatural’ fusion result, it is necessary to extract the main features of layers before the fusion operation.

By extracting the salient information, we make sure that the network pays attention to the most relevant and diagnostically significant details in the input images. For this reason, the salient information for the PCNN input image should be correctly detected.

The structure tensor is valuable because it provides a reliable representation of local patterns. Here, we use the image’s gradient magnitude as the structural tensor. The gradient magnitude represents the rate of pixel intensities change in different orientations. We take advantage of this for leveraging the strength and orientation of edges and corners in the images. This approach enables PCNN to highlight important features that lead to a more effective fusion of MRI and PET/SPECT images. It enhances the network’s ability to focus on salient details, which is crucial in medical imaging applications for accurate analysis and diagnosis.

**Figure 7.**
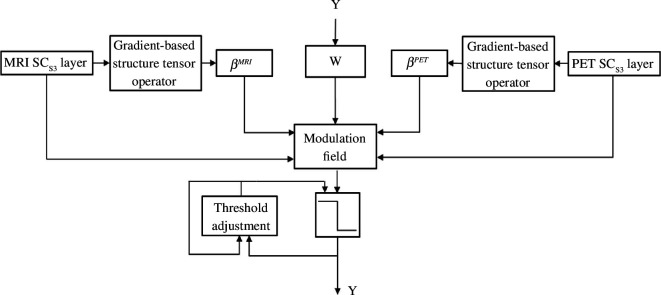
ST–PCNN fusion strategy block diagram.

Since an STS can identify an image’s gradient information efficiently, it make sense to use it to choose structures as the fusion process input. In figure 7, the input image’s salient information is functioning as the joint strength ( $\beta^{MRI}\wedge \beta^{PET}$ ). *Y* is the neuron’s outcome which has an important role in the neuron’s input in each iteration.

A structure tensor is defined as partial derivative information in mathematics. It often represents gradient or edge and corner information in computer vision and image processing, and has a stronger representation of local patterns than the directional derivative due to its coherence measure [33–35]. In other words, a structural tensor uses an image’s gradient magnitude to identify the coordinates of the edges and corners [36].


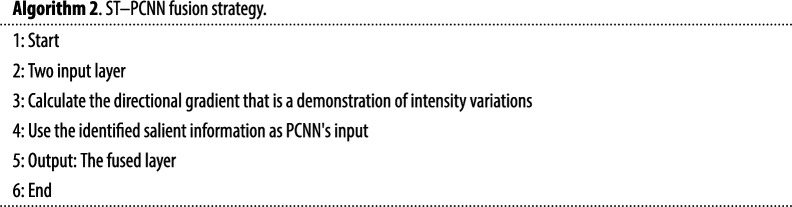


In equations (2.1)–(2.6)–2_2,2_3,2_4,2_5,2_6, ST–PCNN fusion strategy is summarized. $S_{ij}\left(n\right)$ has the pixel value of MRI and corresponding PET image. The input image’s salient information is functioning as the joint strength $\beta^{MRI}\wedge \beta^{PET}$ . *Y* is the neuron’s outcome. $L_{ij}\left(n\right)$ is the linking parameter. $\theta_{ij}$ is the threshold of the step function. $SC_{Fused}\left(x,y\right)$ and $LC_{Fused}\left(x,y\right)$ are final fused SC and LC layers that are generated by ST–PCNN scheme.


(2.1) $$F_{ij}\left(n\right)=S_{ij}\left(n\right),$$

(2.2) $$\beta_{ij}^{MRI}=STS\left(MRI\right)\;\;\;\;\;\;\;\beta_{ij}^{PET}=STS\left(PET\right),$$

(2.3) $$Y_{ij}\left(n\right)=\left\{ \begin{array}{ll} 1 & U_{ij}\geq \theta_{ij}\\ 0 & Otherwise \end{array}\right.,$$

(2.4) $$\begin{array}{l} U_{ij}\left(n\right)=Max\{F_{ij}^{MRI}\left(n\right)\left(1+\beta_{ij}^{MRI}L_{ij}\left(n\right)\right),F_{ij}^{PET}\left(n\right)\left(1+\beta_{ij}^{PET}L_{ij}\left(n\right)\right) \end{array},$$

(2.5) $$T_{ij}=\left\{ \begin{array}{ll} n & U_{ij}\left(n\right)\geq \theta_{ij}\left(n-1\right)\\ T_{ij}\left(n-1\right) & \;\;\;\;\;\;\;\;\;\;\;\;\;Otherwise \end{array}\right.,$$

(2.6) $$SC_{Fused}\left(x,y\right)=\left\{ \begin{array}{ll} SC_{MRI} & T_{xy.MRI}\geq T_{xy.PET}\\ SC_{PET} & \;\;\;\;\;\;\;\;\;\;\;\;\;\;\;Otherwise \end{array}\right.\;\;\;\;LC_{Fused}\left(x,y\right)=\left\{ \begin{array}{ll} LC_{MRI} & T_{xy.MRI}\geq T_{xy.PET}\\ LC_{PET} & \;\;\;\;\;\;\;\;\;\;\;\;\;\;\;\;Otherwise \end{array}\right..$$

#### 2.3.3. Fusion of background component

The background component of an image is the best equivalent of the source images. While the image fusion seeks to merge as much valuable information in the process [18], so the regularly used fusion rule for the background layer is the maximum fusion method [37] equation (2.7).


(2.7) $$BC_{Fused}=max(BC_{MRI},\;BC_{PET}).$$

## 3. Experimental results and analysis

### 3.1. Dataset

For this work, 40 pairs of multimodal images are selected for the evaluation of the proposed methodology. The pair of images includes various case studies including MRI, TITc, SPECT and PET. Each imaging modality reflects different information about human organs and sick tissue. All test images used in this article were gathered from Whole Brain Atlas, which is an online database comprising multimodal nervous system images. This image dataset were created by K. Johnson and J. Becker of Harvard Medical School [38].

### 3.2. Analysis

The suggested algorithm has been experimented with various input image pairs in order to analyse its performance and accuracy. We compared the performance of our proposed framework with the following methods: multi-level morphological gradient (MLMG)-PCNN [16], joint bilateral filter (JBF) [18], Local extreme map Guided filter [39], LRD [7], adaptive co-occurence filter (ACOF) [40], multiple dictionaries and truncated Huber filtering (MDHU) [41], fast guided filtering (FGF) [42] and a fusion method based on phase congruency and local Laplacian energy [43]. In our experiments, subjective and objective evaluation is performed to assess the method’s efficiency. By comparing the output image with the input data images visually, qualitative analysis evaluates the performance of the fusion process. However, quantitative analysis examines the reconstructed images using mathematical modelling [44].

To further enhance the visual comparison between the proposed method and other algorithms, we asked a group of observers to compare our method’s outputs with other methods and score them.

### 3.3. Qualitative analysis

One of the most effective ways to distinguish the performance of medical image fusion methods is to evaluate the fused image by directly using human vision system. The nine-image fusion techniques are compared in this subsection using the qualitative method, which compares the fusion outcomes visually. Throughout this section, we present various examples that highlight the nine-image fusion methods in this section. Intuitively, we may provide some apparent visual judgement from these fusion results.

**Figure 8.**
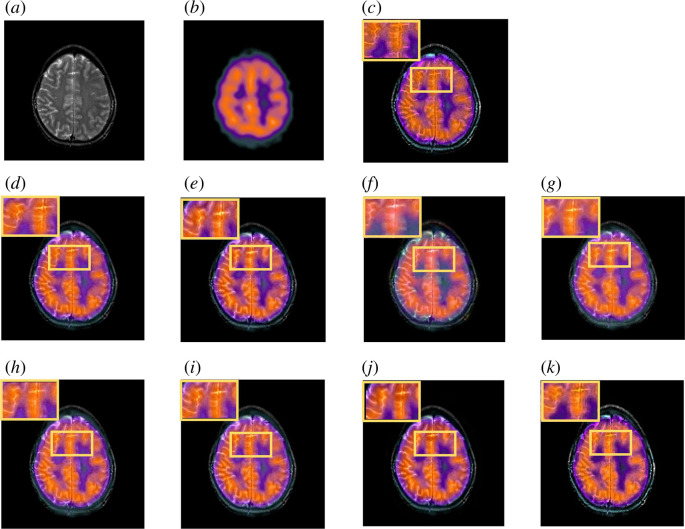
Comparison of fused image on the MRI-SPECT from metastatic bronchogenic carcinoma image dataset. (*a*) Original MRI image. (*b*) Original SPECT image. (*c*) MLMG-PCNN [16]. (*d*) JBF [18]. (*e*) Local extreme map GF [39]. (*f*) LRD [7]. (*g*) ACOF [40]. (*h*) MDHU [41]. (*i*) FGF [42]. (*j*) Fusion based on phase congruency [43]. (*k*) Proposed.

Figure 8 demonstrates the two MRI and SPECT input images from metastatic bronchogenic carcinoma image dataset. Since MRI depicts the brain’s physical anatomical contrast and details, the goal of image fusion is to maintain strong contrast while preserving more structural information. Contrast reduction can be seen in MLMG-PCNN and JBF. On the other hand, SPECT image’s colours have valuable information for the physician. Colour distortion took place in LRD output image. MDHU fused output image has low contrast and lacks sharpness. This suggests that the fusion procedure did not significantly improve the detail’s clarity.

As can be seen, more information might be preserved in the fused output image by using our suggested strategy. Our method outperforms other popular methods in subjective visual perception. The result images have less colour distortion and more structural information included.

**Figure 9.**
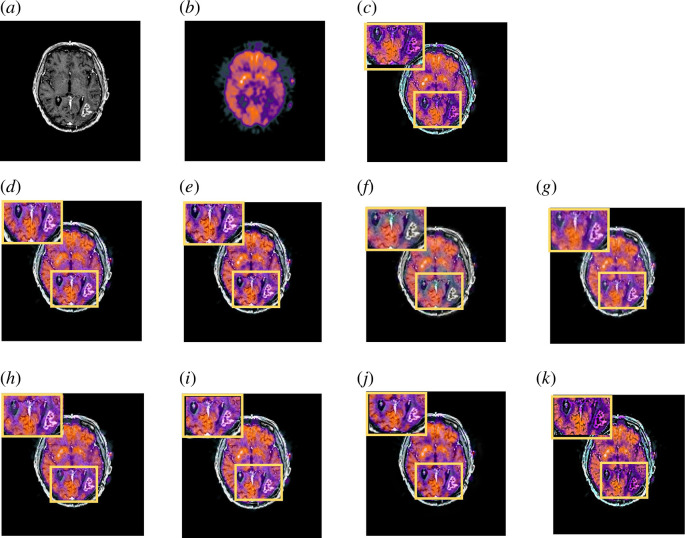
Comparison of fused image on the MRI-PET from Glioma image dataset. (*a*) original MRI image. (*b*) original PET image. (*c*) MLMG-PCNN [16]. (*d*) JBF [18]. (*e*) Local extreme map GF [39]. (*f*) LRD [7]. (*g*) ACOF [40]. (*h*) MDHU [41]. (*i*) FGF [42]. (*j*) Fusion based on phase congruency [43]. (*k*) Proposed.

We have demonstrated comparison examples of the nine-image fusion methods with the two MRI and PET input images from the Glioma image dataset in figure 9. Our proposed method has significantly better visual sharpness than ACOF, MDHU and FGF. Overall, the visual contrast between the structure and the surrounding tissue is increased by the radiological contrasts in our proposed method.

**Figure 10.**
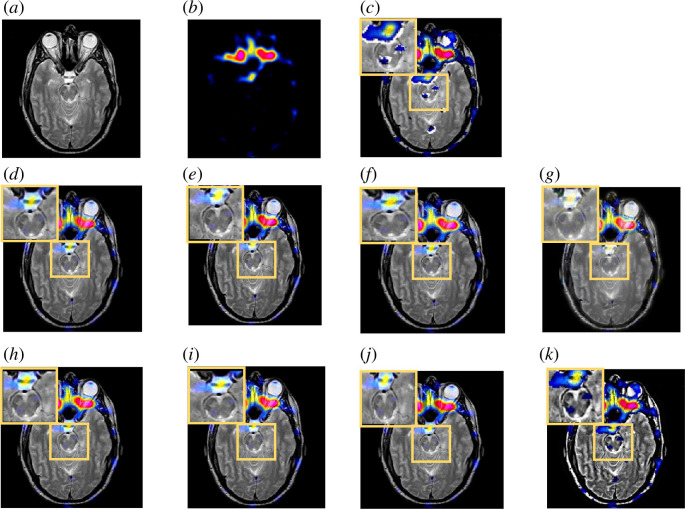
Comparison of fused image on the TITc-SPECT from Glioma image dataset. (*a*) Original MRI image. (*b*) Original SPECT image. (*c*) MLMG-PCNN [16]. (*d*) JBF [18]. (*e*) Local extreme map GF [39]. (*f*) LRD [7]. (*g*) ACOF [40]. (*h*) MDHU [41]. (*i*) FGF [42]. (*j*) Fusion based on phase congruency [43]. (*k*) Proposed.

Figure 10 shows the comparison of fused images on the TITc and SPECT, demonstrating images of neoplastic disease. Our algorithm’s result images have less colour distortion and more structural information included.

**Figure 11.**
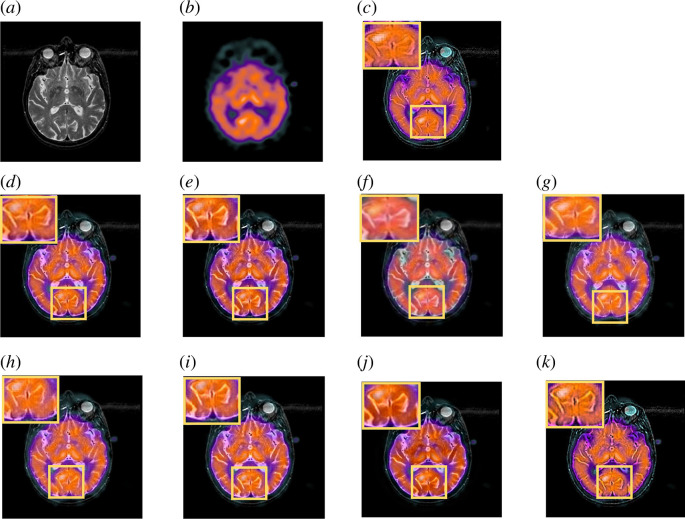
Comparison of fused image on the MRI-SPECT image dataset. (*a*) Original MRI image. (*b*) Original SPECT image. (*c*) MLMG-PCNN [16]. (*d*) JBF [18]. (*e*) Local extreme map GF [39]. (*f*) LRD [7]. (*g*) ACOF [40]. (*h*) MDHU [41]. (*i*) FGF [42]. (*j*) Fusion based on phase congruency [43]. (*k*) Proposed.

In figure 11, LRD and FGF outputs exhibit certain colour changes that hold significant meaning in SPECT images. In SPECT imaging, colour can report important information about the distribution of radiotracers or specific aspects of the brain tissue. This observation emphasizes the importance of preserving the originality of colours and structures in the context of PET/SPECT images. Our method effectively conveys details from the MRI and colours from the SPECT input images compared with other methods. To further enhance the visual comparison between the proposed method and other algorithms, we asked a group of observers to compare our method’s outputs with other methods and score them. Observers are two radiologists from the Royal University Hospital of Saskatoon and all had experience in MRI and PET/SPECT image reading. The radiologists were asked to rate a series of 90 images in four categories independently and pick out which output image they preferred to use for diagnosis. Table 1 demonstrates the scores by human observers. The average scores point out that observers opted to employ our proposed outputs during the diagnostic assessment in two categories. As can be seen, each category of images has different subjective quality evaluation. This is primarily due to the various visual characteristics that each has. Overall, our proposed fusion scheme increased the visual contrast and received positive subjective review.

**Table 1.** Scoring results.

	metastatic carcinoma(MRI-SPECT)	glioma disease(MRI-PET)	normal ageing(MRI-SPECT)	glioma disease(TITc-SPECT)
MLMG-PCNN [16]				X
JBF [18]				
local extreme map GF [39]				X
LRD [7]				
ACOF [40]	X	X		
proposed	X		X	

### 3.4. Quantitative analysis

The implications of image fusion techniques are not only determined by visual analysis but also by numerous academics, who have offered various quality criteria for both qualitative and quantitative studies in order to judge the quality of the output fused image. The objective evaluation results of the fused images are shown in tables 2–5. We examine how well colour and spatial features are preserved. Among the objective assessment indicators are:

1. Entropy, which measures the texture information of the fused image.
2. The sum of correlation of differences (SCD). This quantifies the extent of information collected in the output image [45].
3. Qabf evaluates the noise fusion results and associates visual information with the edge information in each pixel [46].
4. Piella’s metric for measuring the degree of relevant information in the input images that is present in the fused image [47].
5. Qy, a structure-based indicator, analyses how well the original image structural information is retained [48].
6. The SSIM for measuring the fused image’s quality is based on the calculation of brightness, the contrast and the structure terms [49].
7. The amount of information conveyed from the original images to the output fused image is described as feature mutual information (FMI) [50].

**Table 2.** Metastatic bronchogenic carcinoma (MRI-SPECT).

	entropy	Piella SS	FMI	Qy	Qabf	SCD	MSSIM
MLMG-PCNN [16]	4.1755	0.9967	0.8835	0.8345	0.5351	1.2267	0.7959
JBF [18]	4.1752	0.9986	**0.8923**	**0.9384**	0.5608	1.4642	**0.8157**
local extreme map GF [39]	4.1475	0.9865	0.8748	0.8810	0.5207	**1.8883**	0.1518
LRD [7]	**4.4140**	0.9968	0.8682	0.8639	0.4879	1.3548	0.5759
ACOF [40]	4.1985	0.8527	0.8562	0.9043	0.4626	**1.7147**	0.8014
MDHU [41]	4.1874	**0.9984**	0.8673	**0.9325**	0.5068	1.4689	0.8019
FGF [42]	4.2827	0.8858	0.8865	0.9249	0.5358	1.5800	**0.8176**
fusion based on phase congruency [43]	**4.4181**	0.9957	0.8809	0.8136	**0.6455**	1.2868	0.7962
proposed	4.2180	**0.9973**	**0.8879**	0.8835	**0.5632**	1.5769	0.8023

**Table 3.** Glioma disease (MRI-PET).

	entropy	Piella SS	FMI	Qy	Qabf	SCD	MSSIM
MLMG-PCNN [16]	3.1512	0.9987	0.8873	0.8628	0.6207	1.4871	0.8345
JBF [18]	3.0972	**0.9995**	**0.8958**	**0.9554**	0.6803	1.5768	0.8468
local extreme map GF [39]	3.0471	0.9901	0.8749	0.9074	0.5958	1.8915	0.1260
LRD [7]	3.3602	0.9973	0.8812	0.9051	0.5844	1.6828	0.6955
ACOF [40]	3.1631	0.9017	0.8726	0.9396	0.5531	**1.7919**	**0.8482**
MDHU [41]	**4.1964**	0.9984	0.8673	0.9325	0.5068	1.4689	0.8159
FGF [42]	3.2533	0.9993	0.8896	0.9423	**0.6983**	1.6179	**0.8519**
fusion based on phase congruency [43]	3.3243	**0.9995**	0.8896	0.8221	0.6749	1.3797	0.8288
proposed	**3.4445**	**0.9998**	**0.8982**	**0.9560**	**0.6913**	**1.6889**	0.7987

**Table 4.** Normal ageing (MRI-SPECT).

	entropy	Piella SS	FMI	Qy	Qabf	SCD	MSSIM
MLMG-PCNN [16]	4.2270	0.9971	0.8635	0.8117	0.5646	1.0953	0.7686
JBF [18]	4.2562	**0.9993**	**0.8780**	**0.9336**	**0.5983**	1.3738	**0.7918**
local extreme map GF [39]	4.2001	0.9953	0.8616	0.8895	0.5546	**1.8844**	0.1832
LRD [7]	**4.4509**	0.9960	0.8761	0.8888	0.5435	1.4574	0.5793
ACOF [40]	4.2948	0.8457	0.8775	0.8921	0.4645	**1.6664**	0.7880
MDHU [41]	4.2669	0.9991	0.8514	0.9185	0.5455	1.4287	0.7926
FGF [42]	4.3491	0.9991	0.8718	**0.9269**	0.5717	1.5613	**0.7933**
fusion based on phase congruency [43]	**4.4931**	**0.9995**	**0.8802**	0.8312	0.5894	1.1289	0.7752
proposed	4.1989	0.9992	0.8619	0.8939	**0.6161**	1.5548	**0.7933**

**Table 5.** Glioma (TITc-SPECT).

	entropy	Piella SS	FMI	Qy	Qabf	SCD	MSSIM
MLMG-PCNN [16]	4.4374	0.9976	0.8901	0.8790	0.7125	1.5140	0.6704
JBF [18]	4.0876	**0.9995**	**0.9156**	**0.9883**	0.6464	1.5828	0.7207
local extreme map GF [39]	4.5549	0.9981	0.9051	0.9455	0.8200	1.4174	0.2654
LRD [7]	**5.3855**	0.9993	0.8956	0.8518	0.7290	1.3944	0.6479
ACOF [40]	4.0899	0.9772	0.9145	0.9810	**0.8858**	1.6662	0.7162
MDHU [41]	4.0709	**0.9994**	0.9139	0.9827	0.6370	1.6031	**0.7222**
FGF [42]	3.2533	0.9993	0.8896	0.9422	0.6983	**1.7179**	**0.8519**
fusion based on phase congruency [43]	4.3507	**0.9995**	0.9125	0.9471	**0.8699**	1.4116	0.7134
proposed	**4.5912**	0.9983	**0.9193**	**0.9886**	0.8541	**1.6858**	0.6848

Each table shows objective assessment indicators for a separate category of medical conditions. It is worth mentioning that various image categories exhibit different characteristics. That is why we report the average value of 40 pairs of images.

In the comparison tables, each row demonstrates the metrics value for distinguished methods. The two highest values for each set of data are in bold. Considering the results from metastatic bronchogenic carcinoma, glioma disease (MRI-PET) and glioma (TITc-SPECT), results of the proposed method report good entropy, which means higher number of bits need to encode the image’s information.

Specifically, our method achieves a highly noticeable evaluation outcome on FMI, SCD, Qabf and Qy indexes in most categories. This implies that our fusion algorithm not only has better performance in information preservation but also facilitates efficient structural and visual transferring.

### 3.5. Time consumption comparison

It is clear from the objective examination that our method’s fusion results are generally better than the other eight medical image fusion methods. Preserving structural information and conveying a greater amount of information is evident when comparing the evaluation outcomes on FMI, SCD, Qabf and Qy indexes. Our method yields strong performance on other metrics as well.

A metric for evaluating an algorithm’s computation cost is the execution time consumption. Table 6 displays average time cost of each method on the Whole Brain image dataset. In the experiments, all programs were evaluated on the same computation platform MATLAB R2022a on a PC with 2.30 GHz and Intel® Xenon CPU and 16 GB RAM.

**Table 6.** Computational time in seconds.

	metastatic bronchogenic carcinoma	glioma disease	normal ageing	glioma diseaseTITC
MLMG-PCNN [16]	21.7012	22.5147	21.7725	20.1422
JBF [18]	0.5462	0.5633	0.5456	0.5943
local extreme map GF [39]	0.3735	0.3703	0.3827	0.4595
LRD [7]	64.6056	67.2609	64.8531	63.1391
ACOF [40]	0.6078	0.6279	0.5840	0.5879
MDHU [41]	32.5791	38.6330	31.5543	32.8002
FGF [42]	0.7822	0.7816	0.7932	0.7879
fusion based on phase congruency [43]	4.4222	4.4511	4.7147	4.4496
proposed	1.8033	1.7764	1.7549	1.8742

It is evident that, when compared with the other methods, the computation cost of our method is within the intermediate range. Compared with the other comparison approaches, the proposed method is relatively time-efficient in terms of time cost evaluation.

## 4. Conclusion

In this work, we introduce a new fusion approach for better visualization of medical images. It includes three main steps: image decomposition, image fusion and fused image construction. For image decomposition, MLGEPF is proposed that takes advantage of Gaussian filter and a gradient-based Guided filter to extract image sublayers. Also, for the fusion step, a novel gradient domain PCNN based on the structure tensor is used to preserve both the colour information and structure information very well. One of the attractive features of this algorithm is that it iteratively updates the guidance image in the decomposition section, so that the small fluctuations of the input image can be largely smoothed. Accordingly, the salient features extracted by our Guided filter are far more than those extracted by the original Guided filter.

The suggested method and other approaches are examined qualitatively and quantitatively to assess the fusion results. The suggested algorithm is better than other algorithms in both subjective and objective assessments. The comparison results show that the proposed method is the best in the three spectral metrics, FMI, Qy and Qab. Our method yields strong performance on other metrics as well. Moreover, the algorithm is relatively time-efficient in terms of time cost evaluation meaning that the proposed algorithm is applicable for almost real-time utilization scenarios.

## Data Availability

The data that supports the research results of this study are fully available [51]. The dataset underlying the results presented in this article are available in [38].
